# Label-free electrochemical sensor to investigate the effect of tocopherol on generation of superoxide ions following UV irradiation

**DOI:** 10.1186/s13036-018-0099-2

**Published:** 2018-09-12

**Authors:** Li Xia Gao, Chunxiang Bian, Yan Wu, Muhammad Farrukh Nisar, Shida Chen, Chang Ming Li, Ling Yu, Ping Ji, Enyi Huang, Julia Li Zhong

**Affiliations:** 1 College of Bioengineering & School of Life Sciences, Chongqing University, Chongqing, 400044 China; 2 Institute for Clean Energy & Advanced Materials, Faculty of Materials & Energy, Southwest University, Chongqing, 400715 China; 3 Chongqing Municipal Key laboratory of oral diseases and biomedical sciences, Biomedical Engineering of Higher Education, Chongqing, 401147 China; 4 Interdisciplinary Research Centre in Biomedical Materials (IRCBM), COMSATS University Islamabad, Lahore Campus, Lahore, 54000 Pakistan

**Keywords:** Skin, Electrochemical sensing, O_2_^•−^/ROS, UVR, Anti-oxidant screening

## Abstract

**Background:**

Generation of reactive oxygen species (ROS), triggered by ultraviolet radiation (UVR), is associated with carcinogenesis of the skin. UV irradiation induced superoxide anion (O2^•−^) is the key ROS involved in the cellular damage. The cytoprotective efficacy of an unknown anti-oxidant compound can be evaluated by analyzing the production of O2^•−^ from treated cells.

**Methods:**

In this study, a glass carbon electrode functionalized with nanotube@DNA-Mn3(PO4)2 composite was applied to quantitative determination of generation of highly unstable O2^•−^ from the melanoma A375 cell line following UVR(UV, UVA and UVB). In addition, the cytoprotective efficacy of anti-oxidant α-tocopherol was evaluated by quantifying the production of O2^•−^.

**Results:**

The results showed that, UVR triggers generation of O2^•−^ in melanoma A375 cells, and α-tocopherol is effective in diminishing the production of O2^•−^ following UV irradiation. By comparing the conventional cell-survival assays results, we found that our simple and quick electrochemical sensing method can quantify O2^•−^ generation through the biological activity of an anti-oxidant compound (α-tocopherol).

**Conclusion:**

Our label-free electrochemical quantification method for ROS (O2^•−^ major) in cells facing UVR stress demonstrates its potential application for high-throughput screening of anti-oxidation compounds.

## Highlighting points

- A carbon nanotube@DNA-Mn_3_(PO_4_)_2_compositefunctionalized glass carbon electrode was applied for quantitative determination of O_2_^•−^generation from melanoma cell A375 following UV, UVA, UVB irradiation.
- Label-free Electrochemical Sensor was developed to quantify ROS produced in cells exposed to UVR.
- The anti-oxidation efficacy of tocopherol on melanoma cells towards UV, UVA and UVB were also investigated.

## Background

Ultraviolet (UV) irradiation represents one of the most important environmental impacts for humans and recently became prominent because of the depletion of the atmospheric ozone layer, leads to increased UV irradiation exposure by the majority of population [1, 2]. It is well documented that UVR can stimulate the production of a series of ROS [3–5], which may cause cellular oxidative stress injury that is believed to be one of the key factors in carcinogenesis [6, 7]. For the most part, UV light from sunlight consists of three regions of wavelengths: UVC, UVB and UVA. UVC (100–290 nm) is absorbed by ozone (O_3_) in the upper atmosphere but UVB and UVA reach to earth surface, are the major fractions linked to skin diseases. UVB (290–320 nm) is absorbed mostly by the epidermis and keratinocyte DNA, while UVA (320-400 nm) is primarily oxidative in nature and penetrates more deeply into the dermal layers of the skin [8, 9]. Since UV fractions reach different biological layers of skin and lead to skin pathology via different cellular pathways. The effects and mechanisms of action initiated by UVA and UVB have been studied extensively [10–12]. For instance, Petersen et al. reported that superoxide anion (O_2_^•−^) production in HaCaT cells was probably linked to DNA damage induced by UVA [13]. UVB can also induce the formation of ROS, leading to cellular damage [14–16].Among the many ROS species that have been studied, O_2_^•−^ is one of the principal radical species [13, 17–19]. It is generated as a reduced intermediate of molecular oxygen in a variety of biological systems. It can easily form hydroxyl radical (HO^•^) in the presence of transition metal ions such as Fe^2+^ and Cu^2+^ [20]. In addition, the reaction between O_2_^•−^and nitric oxide (^•^NO) leads to the formation of highly reactive peroxynitrite (ONOO-) in the pathogenesis of atherosclerosis and neurodegenerative diseases [21]. As a consequence, efforts have been made to investigate generation of O_2_^•−^ induced by UVR. Particularly, the cytoprotective efficacy of an unknown anti-oxidant compound can be evaluated by analyzing the production of O_2_^•−^ from treated cells.

A survey of the literature shows that the main techniques for measuring O_2_^•−^ are based on probe-labelling assays. Intracellular fluorescent histochemistry [22], flow cytometry [16] and spectrofluorometric analyses [23, 24] are the most used approaches to characterize ROS such as O_2_^•−^ by using fluorescent dyes such as 2′,7′-dichlorofluorescein diacetate (DCFH-DA), hydroethidine and dihydrorhodamine [1, 23]. The production of extracellular O_2_^•−^ can also be measured by using the ability of O_2_^•−^ to reduce ferricytochrome C that was added to the cell suspension [25]. Another advanced technique is electron spin resonance (ESR)-spin trapping, was applied for determination of O_2_^•−^ generated by UV-irradiated skin cells [10, 24]. Apart from the expensive equipment and complicated assay procedures, these probe-labelling approaches are time-consuming, difficult to automate and highly prone to interference. The short lifetime of free radicals, such as O_2_^•−^, particularly demands fast response of the analytical tool to the changes in concentration to obtain sufficient signal-to-noise ratios [20, 26]. Electrochemical biosensors have become promising candidates for real-time analysis of free radicals, since they provide the advantages of rather simpler equipment and operation protocols. Li et al. [20] found that an electrochemical biosensor can sense O_2_^•−^ released from cancer cells, using potassium-doped multi-walled carbon nanotubes (KMWNTs)-1-butyl-3-methylimidazoliumhexa-fluorophosphate ionic liquid composite gels [20]. While hydrogen peroxide (H_2_O_2_) can directly be measured in cells growing in a 3D matrix environment [27]*.* These studies indicate that, with intimately coupled biological recognition elements and electrochemical transduction units, an electrochemical biosensor exhibits great potential for facilitating understanding of biological process. Also, the use of a small-volume sample allows expensive reagents, particular for rare clinical biopsy samples, to be conserved and makes the analysis more cost-effective.

In this study, we have designed and developed a label-free electrochemical sensor that can measure the generation of O_2_^•−^ from UVR exposed melanoma A375 cells, though other ROS species e.g. H_2_O_2_ has not been totally excluded. To demonstrate the electrochemical analytical power of this sensor, the protective effects of model anti-oxidant (α-tocopherol) was studied in melanoma A375 cells. The specific objectives to be addressed were [1] to measure generation of O_2_^•−^ from melanoma A375 cells following exposure to UVR alone i.e., UV, UVA and UVB; [2] to quantify the production of O_2_^•−^ from cells pre-treated with α-tocopherol following UVR irradiation; [3] to compare the data from electrochemical measurement, the cell-survival assay and conventional ROS fluorescence staining therefore establish the potential use of the label-free electrochemical method for high-throughput screening of antioxidants.

## Methods

### Materials

The human melanoma cell A375 (purchased from ATCC) were maintained in RPMI 1640 medium (Gibco®) supplemented with 10% foetal calf serum (FCS, Gibco®) with 100 U mL-1 penicillin and 100 U mL-1 streptomycin at 37 °C in a humidified 5%CO_2_ incubator. α-Tocopherol was purchased from Sigma-Aldrich. 3-[4,5-Dimethylthiazol-2-yl]-2,5-diphenyltetrazolium bromide (MTT), 2-(4-amidino¬phenyl)-6-indole carbamidine dihydrochloride (DAPI), 2′,7′-dichlorodihydrofluorescein diacetate (DCFH-DA) and superoxide dismutase (SOD) were purchased from Beyotime Biotechnology (Beijing, China). All other chemicals were purchased from Sigma-Aldrich and were used without further purification, unless otherwise indicated. All solutions were prepared with deionized (DI) water produced by a PURELAB flex system (ELGA Corporation).

### Apparatus

#### UV irradiation

Cells were irradiated with a broad-spectrum UV lamp (MUA-165, Japan). The proportion of UVA and UVB in delivered UV light from this lamp is around 20:1*,* a ratio close to solar light at mid-day [28]. The lamp exposure time was calculated using an ultraviolet radiometer (Photoelectric Instrument Factory, Beijing Normal University, China). Appropriate glass filters to block UVA, UVB were used respectively to provide fractions of UVA and UVB in the following experiments, according to previous studies [29–33].

#### Electrochemical detection setup

**Fig. 1:**
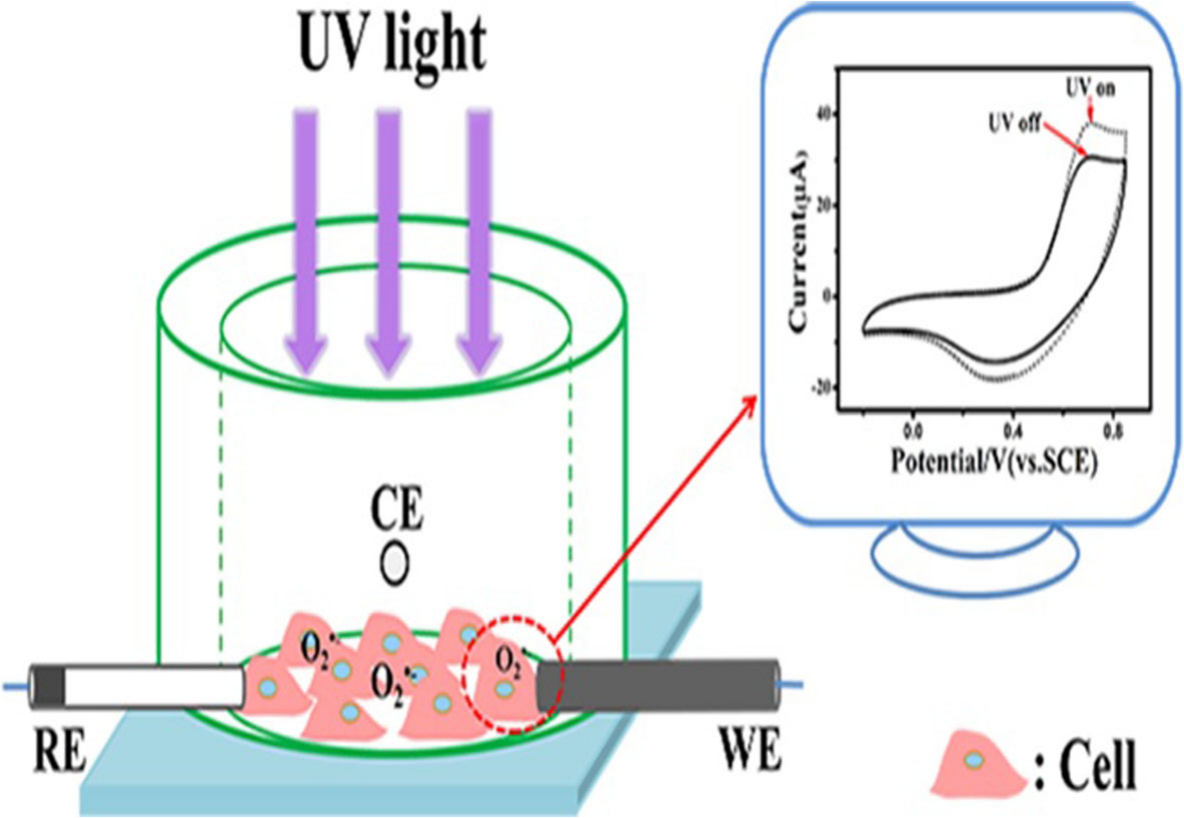
Electrochemical sensor for quantifying generation of superoxide anion (O_2_^•−^) from cells during UV irradiation. UV: ultraviolet; RE: reference electrode; CE: counter electrodes; WE: working electrode

The experimental setup is shown in Fig. 1. A three-electrode system was employed, with a nano material-functionalised glass carbon electrode (GCE) as a working electrode (WE), an Hg/HgCl_2_/KCl electrode as a reference electrode (RE) and a platinum wire as a counter-electrode (CE). To avoid interference from direct UV irradiation of the electrodes, the electrodes were assembled on the side-wall of the detection well. In addition, a piece of aluminium foil with a hole was placed on top of the detection well to confine the UV light to impacting only on the cells. Electrochemical detection was carried out with an electrochemical station (CHI 760e, Chen Hua Instruments Co. Ltd., China).

### Electrochemical detection of O_2_^•−^ released by the cells

#### Preparation of an O_2_^•−^ electrochemical sensor

The construction of electrochemical sensor for O_2_^•−^ detection was detailed in our recent paper [34, 35]. In brief, single stranded DNA (2.1 mg) was added to MnSO_4_ (1.0 mL, 0.10 M) under constant stirring at 60°Cstirring until the solution became transparent. The pellet of DNA-Mn_3_(PO_4_)_2_ composite was recovered by centrifugation. To prepare the O_2_^•−^ sensing electrode, carbon nanotube (CNT, 0.5 mg mL^− 1^) and DNA- DNA-Mn_3_(PO_4_)_2_ (0.20 M) were drop-casted on a polished glass-carbon electrode. To detect O_2_^•−^, electrodes modified with CNT@DNA-Mn_3_(PO_4_)_2_ composite were exposed to aliquots of KO_2_ and cyclic voltammetry curves were recorded. The peak current value at 0.70 V was recorded and was plotted against the concentration of KO_2_to draw a current-dose calibration curve.

#### In situ detection of production of O_2_^•−^ from UV-irradiated cells

Confluent A375 cells were seeded into 24 cell plates, at a density of 1 × 10^5^ per well, and were incubated for 6 h. Then α-tocopherol (25 μM was chosen due to optimize concentration [33]) was added into cells and incubated for another 12 h. The cell-culture disc with adherent cells was transferred to the electrochemical detection cell (Fig. 1), to which serum free medium (2.5 mL) was added. Then the cells were exposed to UVR and the electrochemical signal arising from O_2_^•−^ were recorded by CHI. Cells without anti-oxidant were measured with the same schedule as controls.

### Fluorescent staining of oxygen radicals

#### Intracellular ROS

A375 cells were collected and seeded at a density of 1 × 10^5^ per well in 24-well plates and were incubated overnight with or without α-tocopherol. Intracellular generation of ROS upon UV irradiation was determined with a DCFH-DA (10 mM) assay kit (Beyotime Inc. S-0033). In brief, following UVA irradiation, cells are washed twice in phosphate buffer saline (PBS) and allowed to incubate in 10 μM/L DCFH-DA (1:1000 dilution in PBS) at 37 °C for 20 min according to the manufacturer’s instructions (Beyotime Inc.). The excess DCFH-DA was rinsed off with phosphate buffer saline (PBS) (1.0 mM). The cells were then observed and imaged under a fluorescence microscope (IX-71, Olympus Corp., Tokyo, Japan).

#### O_2_^•−^colorimetric assay kit

Superoxide assay kit (S-0060 Beyotime Biotechnology Beijing Inc.) was conducted following the product instructions. In brief, A375 cells were seeded (1 × 10^4^) in 96-well plates and 80–90% confluence when treated. Now the cells were washed three times in PBS, and allowed to incubate in 10 μmol/L DCFH-DA (1:1000 dilution) at 37 °C for 3 min (Beyotime Biotechnology, Beijing Inc.). The excess DCFH-DA was rinsed off with PBS (1.0 mM). Now the cells were irradiated with respective UVR (UV, UVA or UVB) stimulus. Prepare the three wells without irradiation should be used as a blank control. Select 2 wells to add 2% SOD (Beyotime Biotechnology, Beijing Inc.) in the wells of the stimulus to verify the entire assay system. The cells were then observed and imaged under a fluorescence microscope (IX-71, Olympus Corp., Tokyo, Japan).The absorbance values taken at 450 nm were compared with the reference absorbance values at 630 nm following to manufacturer’s instructions. These data were expressed as the percentage of increase of absorbance value compared to sham controls (sham-irradiated groups).

### Cell viability assay

The protective effects of α-tocopherol on UV-irradiated melanoma cell A375 were evaluated using the MTT assay. In brief, cells were seeded at a density of 1 × 10^4^ per well in 96-well plates and incubated with or without the α-tocopherol for 12 h. Following challenge with UVR, the cells were cultured in conditional culture medium for a further 24 h. Each well then received 10 μL of MTT solution (0.5 mg mL^− 1^) and were incubated for 3–4 h at 37 °C. Then 100μLlysisbuffer (mixture of 2-propanol, Triton-100 and hydrochloric acid) was added to each well to dissolve the purple-coloured formazan produced by viable cells, before colorimetric analysis. The absorbance at 570 nm was measured by a microplate reader and the results were expressed as the percentage of surviving cells compared to the sham-irradiated vehicle control group.

### Statistical analysis

Results are expressed as means±standard error of the mean (SEM). The data were analysed by Student’s t-test using Origin Statistic software (Origin Lab Corporation, USA). *P* < 0.05 was considered significant. All experiments were performed three independent times in triplicates.

## Results and discussion

### Quantification of UV-inducedO_2_^•−^ in melanoma cells by label-free electrochemical sensor

**Fig. 2:**
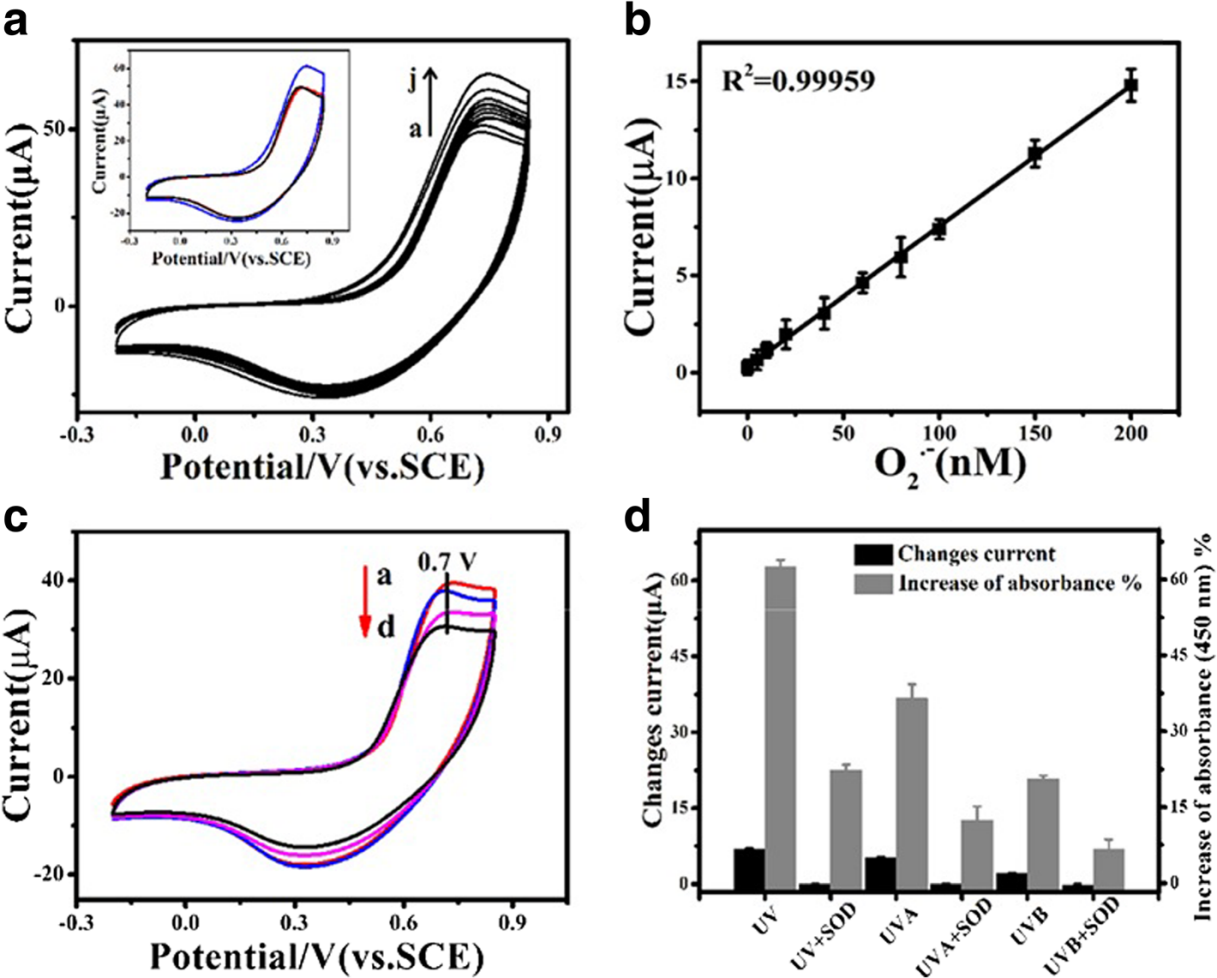
Calibration of CNT@DNA-Mn_3_(PO_4_)_2_ modified glass-carbon electrode for measurement of O_2_^•−^ and electrochemical characterization of O_2_^•−^ released by cells following UVR irradiation. **a** Cyclic voltammetry (CV) curves measured with PBS and PBS plus different concentrations of KO_2_ (nM). *a*: 0, *b*: 5, *c*: 10, *d*: 20, *e*: 40, *f*: 60, *g*: 80, *h*: 100, *i*: 150, *j*: 200; inset: CV curves measured with PBS (black), PBS plus KO_2_ (150 nM, blue), and KO_2_ solution added with SOD (300 U mL^− 1^, red). **b** Calibration curve for serial concentrations of O_2_^•−^. **c** O_2_^•−^ CV curves of cells under irradiation, *a.* UV, *b*. UVA, *c*. UVB, *d*. Sham-irradiation control. **d** Histogram of peak current changes compared to sham-irradiated cells (black column, *n* = 3) and generation of O_2_^•−^ upon irradiation with UV as quantified by the O_2_^•−^assay kit: increase of absorbance at 450 nm (grey column, n = 3). SOD: superoxide dismutase. UV (10.5 J cm^− 2^ = 105 kJ m^− 2^), UVA (10 J cm^− 2^ = 100 kJ m^− 2^), UVB (0.5 J cm^− 2^ = 5 kJ m^− 2^)

To evaluate the feasibility of using an electrochemical method to quantify production of O_2_^•−^ induced by UV irradiation from melanoma cells, an electrochemical sensor was fabricated and characterised. Initially, cyclic voltammetry (CV) was performed with PBS to characterize the CNT@DNA-Mn_3_(PO_4_)_2_ functionalised electrochemical sensor. KO_2_ was used as the source of O_2_^•−^ as reported in literature [19]. The peak current of the oxidative state/stress at 0.7 V increases when KO_2_ was added to PBS (blue line inset of Fig. 2a). The production of superoxide anions (O_2_^•−^) is linked with diverse range of biochemical phenomenon leading to various oxidative stress mediated pathologies. The electrochemical determination of superoxide ion is solely depending on SOD enzyme, hence check it’s reproducibility as well as not a cost effective, and advancement in nanosciences solved certain limitations with this procedure. Previously we demonstrated that Mn_3_(PO_4_)_2_ nanosheets which are a biomimetic enzyme were template-synthesized with DNA and further assembled on carbon nanotubes (CNTs) that form nanocomposite sheets (DNA-Mn_3_(PO_4_)_2_-CNT) [34]. The dismutation of superoxide ion was catalyzed by Mn_3_(PO_4_)_2_ sheets, but the CNTs allows quick shifts of electrons to maximize its sensitivity, specificity and reproducibility for O_2_^•−^ detection [34].

The increased peak current gradually decreased to PBS level after the introduction of SOD to decompose/neutralize O_2_^•−^ (red line inset of Fig. 2a). Different concentrations of KO_2_ were added to vary the amount of O_2_^•−^ and the peak current at 0.7 V was recorded that was plotted against the concentration of O_2_^•−^. The corresponding calibration curve of current response vs. O_2_^•−^ concentration showed a sensitivity of 70 nA nM-1 (Fig. 2b).The CV curves showed that, the change in current (μA) is associated with changing in the production of O_2_^•−^ at different UV treatments (UV, UVA, UVB) and in sham-irradiated control (Fig. 2c). Furthermore, to verify the electrochemical-sensing efficacy, the O_2_^•−^ assay kit was used to measure O_2_^•−^ generated from melanoma cells challenged with UVR; a similar increase was observed in both methods. Incubation of cells with SOD eliminatesincreasedO_2_^•−^ in the culture medium, while the electrochemical sensing measures a similar pattern of generation of UVR-induced O_2_^•−^as did conventional fluorescent staining for O_2_^•−^ (Fig. 2d), the former with less consumption of sample and shorter experimental time. These suggested that, the electrochemical sensor provides a possibility of recording production of O_2_^•−^ in groups of A-375 cells facing different doses of UVR, without disturbing the cellular metabolism.

### Measurement of UV-irradiation induced O_2_^•−^ and its diminution with α-tocopherol by using label-free electrochemical sensor

To demonstrate the feasibility of electrochemical sensor as an analytical tool to evaluate the anti-oxidative capability of compounds, we pre-treated UVR-challenged cells with an antioxidant compound (α-tocopherol). The electrochemical sensor was used for direct measurement of O_2_^•−^ generated from A375 cells pre-treated with α-tocopherol followed UV irradiation. In addition free-radical-scavenging effects of the antioxidant α-tocopherol in A375 cells given UVA, UVB and bulk UV were also investigated with the same electrochemical sensor. For this purpose, the normalized current change at 0.7 V was calculated in sham-irradiated cells as a control by using following equation.

1 $$ \boldsymbol{\Delta}\ \mathbf{C}\mathbf{urrent}\%=\left[\left(\mathbf{Ci}-\mathbf{C}\mathbf{0}\right)/\mathbf{C}\mathbf{0}\right]\times \mathbf{100}\% $$

Where Ci is current value measured upon UV irradiation, C0 is current value of sham control (sham-irradiated).

**Fig. 3:**
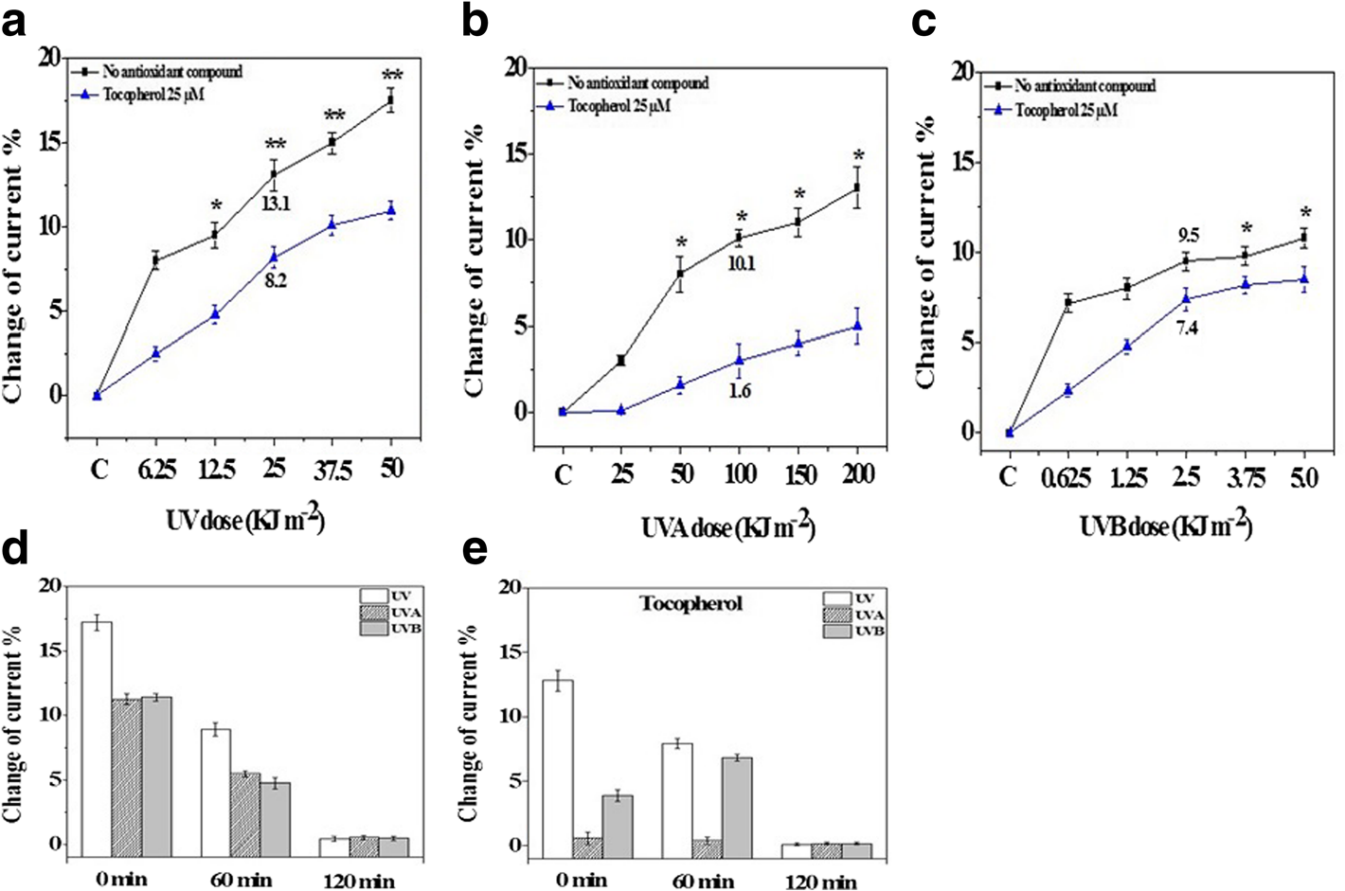
**a-c** Extracellular O_2_^•−^ quantified by electrochemical method in melanoma A375 cells. A375 cells were exposed to UV (A), UVA (**b**) and UVB (**c**); Non-pretreatment: black line; α-tocopherol pre-treatment: blue line. O_2_^•−^ generation during post-UVs irradiation quantified by electrochemical sensing: non-treatment (**d**) α-tocopherol pretreatment (**e**) (*n* = 3),* denotes *P* < 0.05,** denotes *P* < 0.01

The Δ Current % was plotted against irradiation energy dose of UV fractions. A higher change in current indicated a higher generation of O_2_^•−^when compared with sham-irradiated control. The changes of production of O_2_^•−^ in melanoma A375 cells irradiated with UVs (UV, UVA, UVB) was comparatively measured by the electrochemical sensor (Fig. 3a-c). Different UV doses (0–105 kJ m^− 2^) induced the production of O_2_^•−^, that altered the currents by 13.1% (50 kJ m^− 2^) to that of the sham. Tocopherol (25 μM) treatment to UV-challenged cells reduces the production of O_2_^•−^ to 8.2% (50.25 kJ m^− 2^) when compared to UVs-irradiation only (Fig. 3a). Similarly, pre-treatment of cells with tocopherol efficiently decreased (8.5%) the production of O_2_^•−^ induced by a UVA (100 kJ m^− 2^) dose to 1.6% compared to sham (Fig. 3b). In contrast, pre-treatment of cells with α-tocopherol followed by UVB irradiation, showed little effects on the production of O_2_^•−^ (Fig. 3c).

The electrochemical sensor is not only able to quantify the generation of O_2_^•−^ at acute phase following UV-irradiation, but also enabled quantification at post-UVR challenge period. After 60 min. Following UVR, in melanoma A375 cells O_2_^•−^ generated current was decreased to half (8.5%) than at zero time point (17%), while at 120 min. The change in electric current faded to the background level (Fig. 3d).

The effect of UV on O_2_^•−^ production are not the sum of the total effects of UVA and UVB, the production of O_2_^•−^ from cells challenged by various UV doses (0-105 kJ m^− 2^, Fig. 3a) is lower than the sum of changes induced by UVA (Fig. 3b) plus UVB (Fig. 3c). Next, we found that α-tocopherol plays a distinct role and lessen the generation of O_2_^•−^ in pre-treated A375 cells followed by UV irradiation, however, UVA and UVB showed different capacities to trigger the generation of O_2_^•−^in melanoma A-375 cells.

The pre-treatment of α-tocopherol reduced the UVA and UVB induced O_2_^•−^ generation by almost 85% and 20%, respectively (Fig. 3b,c). This difference may due to the generation of H_2_O_2_, or interference of some other unknown reason yet, hence need further optimisation of the method. Furthermore, antioxidant tocopherol showed different behaviour against UVA and UVB induced O_2_^•−^ is due to the difference in wavelength energy. In addition to variable UV wavelength effects, antioxidants are distinct chemical units with different modes of action for their effects and possess a unique biochemical profile hence making it different from other related compounds. Based on the results of electrochemical sensor, we speculated that α-tocopherol showed different protective properties in melanoma cells exposed to different UV wavelengths. In general, the electrochemical sensor is able to characterize the O_2_^•−^ production, as cellular oxidative status is one of the indicators.

### Prediction of protective effect of α-tocopherol on cell survival through electrochemical sensing

To analyse the effect of α-tocopherol on cell survival following UVR, A375 cells were exposed to different doses of UV, UVA and UVB, and the anti-oxidant effect of α-tocopherol was assessed by calculating the cell survival rate by using following equation.

2 $$ \mathrm{Cell}\ \mathrm{survival}\%=\left[\left({\mathrm{A}}_{\mathrm{T}}\hbox{--} \mathrm{Ac}\right)/\mathrm{Ac}\right]\times 100\% $$

**Fig. 4:**
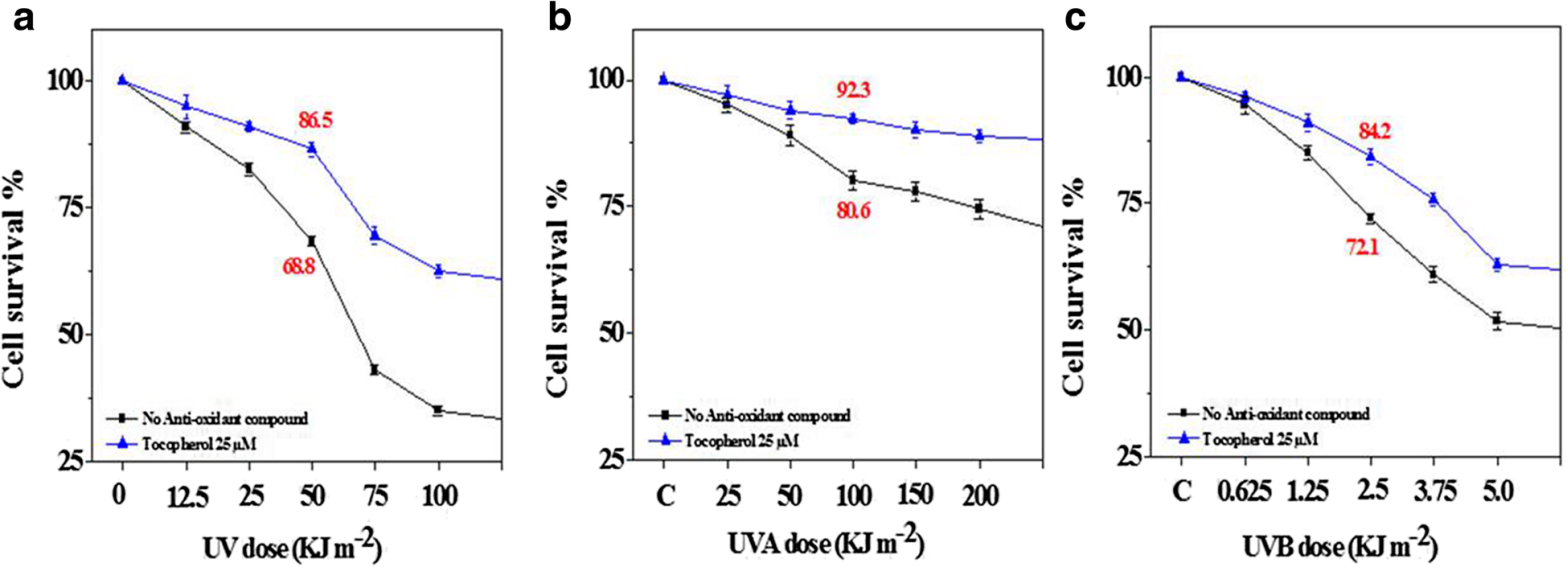
Survival of cells pre-treated by α-tocopherol under UV irradiation with melanoma A375 cells. These survival rates were calculated using sham-irradiated cell as reference, (*n* = 3). (**a**) UV dose (50 kJ m−2) decreases cell survival to 68.8% with α-tocopherol recurrence of 86.5% cell viability. (**b**) Equivalent UVA dose (100 kJ m−2) gave cell viability loss to 80.6% which is recovered by α-tocopherol to 92.3% cell viability. (**c**) Finally the equivalent UVB dose (2.5 kJ m−2) given cell viability of 72.1% that finally recovered by α-tocopherol to 84.2% viable cells. Non-pretreatment: black line; α-tocopherol pre-treatment: blue line

Where A_T_ is the absorbance value of UV-irradiated cells and Ac is absorbance value of sham-irradiated cells. This calculation, gives survival of cells under UV irradiation is normalized to the sham or control cells. The α-tocopherol had significant cytoprotective effects on melanoma A375 cells, and it enhanced cell survival (18%) following UV light exposure (50.25 kJ m^− 2^ = UVA 50 kJ m^− 2^ + UVB 0.25 kJ m^− 2^) (Fig. 4a). During studies of UVA-induced cell damage, α-tocopherol appeared to be more cytoprotective as indicated by the survival rates of pre-treated (92.3%) compared with control melanoma A375 cells (80%), with 10% marked and significant difference in reduction of cell survival (Fig. 4b). On the other hand, UVB induced cell viability loss (72%) was significantly repaired by α-tocopherol pre-treatment (84%) compared with control, with 12% of protection revival (Fig. 4c).

Collectively, MTT cell-survival assay showed that α-tocopherol is effective in diminishing UV, UVA and UVB-induced damage in melanoma A375 cells, this is similar to results given by electrochemical sensing and colorimetric assay for O_2_^•−^ levels (Fig. 3a-e). Interpreting the survival rate (Fig. 4a-c) of cells pre-treated with α-tocopherol followed by UV irradiation caused the generation of O_2_^•−^ (Fig. 3a-c) may efficiently harbour the oxidative stress and lessen it promptly. The different cytoprotective efficacy of α-tocopherol against UVA- or UVB-induced cell death was decreased, thus could be specified as the distinct capability of α-tocopherol to eliminate UV fraction induced oxidative stress, since UVA is the major oxidative stress causing wavelength in UV.

### Comparison of methodologies towards UV-induced cellular oxidative stress

The MTT assay was conducted to evaluate the loss of viability induced by UV irradiation. It monitors the overall cell growth over a period of time, thus showing its strength in evaluating the long-term effect of a treatment on cells [34, 35]. A large body of work, spanning more than four decades, demonstrates fluorescent probe labelling for characterisation of changes of intracellular ROS [36, 37].

**Fig. 5:**
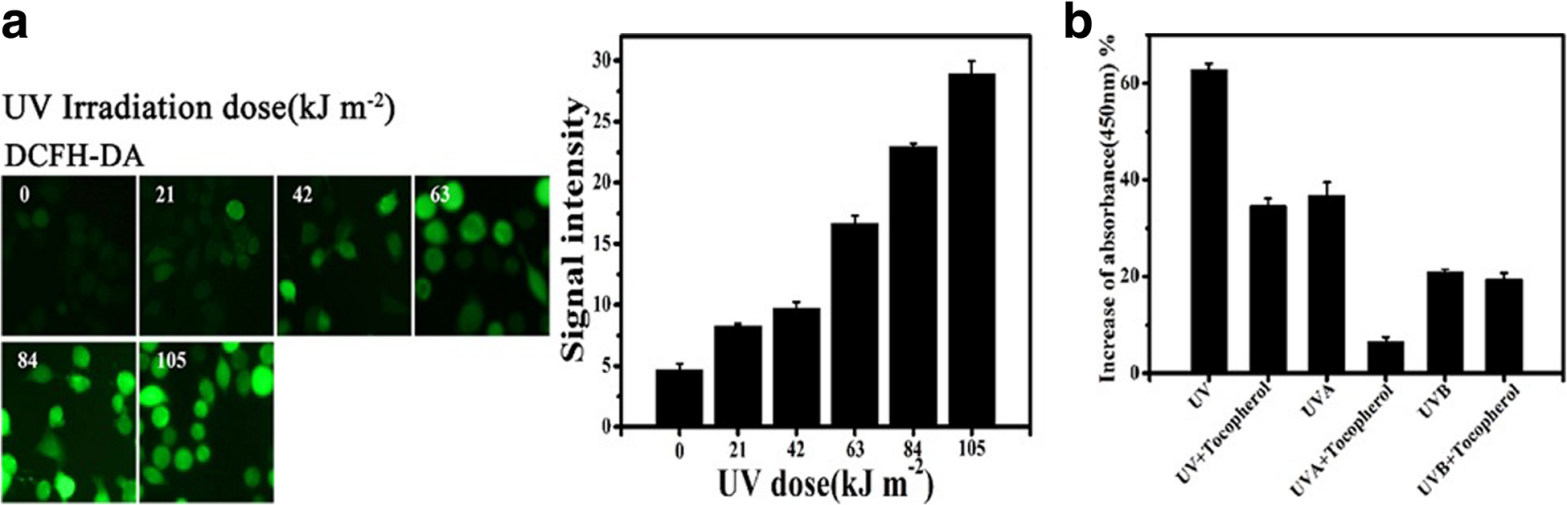
Fluorescent characterisation of intracellular ROS production and DNA damage in UV irradiated A375 cells. **a** Fluorescent microscopy images of DCFH-DA staining and histogram of fluorescent intensity of DCFH-DA staining quantified by Image J, *n* = 3. **b** Generation of ROS (O_2_^•−^ major) from A375 cells quantified O_2_^•−^ colorimetric assay kit, *n* = 3

However, it is also clear that this probe-labelling method measures transient fluxes of oxidants only with great difficulty [38, 39]. Most importantly, the labelling of cells challenges the possibility to characterize the changes in situ of cells during a time period in responding to a treatment. The merits of label-free electrochemical sensing have been discussed in the literature [27, 34, 40] (Table 1). To confirm the feasibility of electrochemical sensing as an analytical tool to determine the anti-oxidative capability of unknown compounds, we performed conventional O_2_^•−^ fluorescent staining with a standard DCFH-DA fluorescent staining protocol that measures intracellular ROS from cells after exposure to UV light. It is clear from the results that UV irradiation can induce an increase of the intensity of the signal for green fluorescence. Figure 5a presents the quantitative changes of ROS (O_2_^•−^) measured by fluorescent staining of three independent methods. The fluorescence signal is gradually increased in response to increasing UV dose and the maximum signal is given by cells irradiated with 105 kJ m^− 2^ UV light. Increases of intracellular ROS following UV irradiation visualized in this study are well in line with previously reported studies [17, 23] and our present electrochemical sensing results. Further increase of UV energy (21 J cm^− 2^ = 210 kJ m^− 2^) does not give further increased ROS fluorescence signal, perhaps due to significant cell death (Data no shown).

**Table 1:** Comparison of methods used to study effect of UV irradiation induced oxidative stress

	MTT assay	Fluorescent assay	Electrochemical detection
Labeling	Yes	Yes	No
Real time	NO	NO	Yes
Sample consumption	Large	Large	Small
Assay time	24–72 h	1-2 h	Within minutes
Overall comparison	Alive cell catalyse enzyme to indirectly characterize cell growth. Evaluate the overall effect of treatment on cell survival, growth.	Fluorophore probe is used to label target molecules. Not feasibly for real-time characterizing a reaction. Staining process will affect the health of cells.	In site characterize a reaction. No damage to cells. Fast reaction time allows for high throughput screening. No expensive equipment is involved.

The protective efficacy of anti-oxidant α-tocopherol was assessed and much attention was paid to the UV dose of 105 kJ m^− 2^, i.e. [UVA (10 J cm^− 2^) + UVB (0.5 J cm^− 2^)]. The O_2_^•−^ assay kit was used to measure the colorimetric changes induced by O_2_^•−^ in a 96-well plate experimental setting. The results showed that the anti-oxidant can reduce the generation of O_2_^•−^ induced by UV in A375 cells. Different anti-oxidation efficacies of the model anti-oxidant are observed in melanoma A375 cells against UVA and UVB exposure (Fig. 5b). It is encouraging to see that standard labelling techniques and electrochemical quantification depict a response pattern of UV irradiation and the anti-oxidant effects of α-tocopherol which are well correlated. In addition, electrochemical sensing revealed the oxidation status, which can be used to predict UV-induced ROS efflux and ultimately cell damage. The fast and easy experimental procedures permitted us to test the efficacy of anti-oxidant α-tocopherol against UVA and UVB in melanoma cells [33, 40]. We demonstrate the potential of electrochemical methods for high throughput screening of potential anti-oxidant compounds, although further improvements in the signal-to-noise ratio are needed. We believe that electrochemical sensing would be a potent and economical candidate to provide meaningful complementary information for standard fluorescent staining experiments.

## Conclusions

The results showed, in melanoma A375 cells electrochemical sensing both UVA and UVB -induced production of ROS e.g. O_2_^•−^. The potential use of in UV (UV, UVA, UVB) involved biological research is enhanced, since the electrochemical sensor quantified O_2_^•−^ levels in medium could be correlated with the cell survival ability under the protection of anti-oxidant α-tocopherol as well. The electrochemical changes occurred with O_2_^•−^ production, can be seen with this label-free method correlated with intracellular fluorescent staining as well. It highlighted the potential of the electrochemical method for high-throughput screening of anti-oxidant under UV irradiation, and for investigation of their related mechanisms.

## Abbreviations

DCFH-DA: 2′,7′-dichlorodihydrofluorescein diacetate
H_2_O_2_: Hydrogen peroxide
kJ m^− 2^: Kilo Jouls per square meter
MTT: 3-[4,5-Dimethylthiazol-2-yl]-2,5-diphenyltetrazolium bromide
O2^•−^: Superoxide anion
ROS: Reactive oxygen species
SOD: Superoxide dismutase
UVA: Ultraviolet A
UVB: Ultraviolet B
UVR: Ultraviolet radiation
